# Discovery of an Auto-Regulation Mechanism for the Maltose ABC Transporter MalFGK_2_

**DOI:** 10.1371/journal.pone.0034836

**Published:** 2012-04-17

**Authors:** Huan Bao, Franck Duong

**Affiliations:** Department of Biochemistry and Molecular Biology, Faculty of Medicine, Life Sciences Institute, University of British Columbia, Vancouver, British Columbia, Canada; University of Queensland, Australia

## Abstract

The maltose transporter MalFGK_2_, together with the substrate-binding protein MalE, is one of the best-characterized ABC transporters. In the conventional model, MalE captures maltose in the periplasm and delivers the sugar to the transporter. Here, using nanodiscs and proteoliposomes, we instead find that MalE is bound with high-affinity to MalFGK2 to facilitate the acquisition of the sugar. When the maltose concentration exceeds the transport capacity, MalE captures maltose and dissociates from the transporter. This mechanism explains why the transport rate is high when MalE has low affinity for maltose, and low when MalE has high affinity for maltose. Transporter-bound MalE facilitates the acquisition of the sugar at low concentrations, but also captures and dissociates from the transporter past a threshold maltose concentration. In vivo, this maltose-forced dissociation limits the rate of transport. Given the conservation of the substrate-binding proteins, this mode of allosteric regulation may be universal to ABC importers.

## Introduction

ATP binding cassette (ABC) transporters utilize ATP to transport a wide range of substrates across cellular membranes [1]. ABC transporters are typically made of two nucleotide-binding domains and two transmembrane domains that alternate into two distinct conformations: Inward facing (P-closed) and outward-facing (P-open). This ATP-driven alternate conformational change allows the capture of the substrate on one side of the membrane and its release to the other side; the so-called alternate access model [2]. For ABC exporters, which include drug, lipid and (poly) peptide transporters, the substrate itself suffices to trigger the ATPase [3]. For ABC importers, as found in bacteria to acquire nutrients, the transport activity depends on a substrate-binding protein located in the extra-cytosolic side of the membrane [4]. The protein usually consists of two symmetrical lobes that rotate toward each other to capture the substrate with high-affinity; so-called closed-liganded conformation [5]. In the maltose transport system, certain mutations in MalFGK_2_ render transport independent from the maltose-binding protein MalE. In that case, translocation of maltose is strongly reduced as the ½ maximal rate of transport (*K_t_*) increases from 2 µM to 1 mM [6]. The function of MalE is therefore essential to increase the affinity of the transporter for the substrate, and therefore the efficiency of transport.

Since MalE is found soluble in the periplasm, it has naturally been proposed that the protein shuttles back-and-forth to the membrane to deliver maltose. The reconstitution of the reaction in proteoliposome and the crystallographic analysis of the transporter have completed the model [7]–[10]. Upon binding of closed-liganded MalE, MalFGK_2_ switches toward the P-open conformation. This structural change forces the opening of MalE and the subsequent release of maltose inside the transporter cavity. After ATP hydrolysis, MalFGK_2_ returns to the P-closed state, maltose is released in the cytosol, and MalE returns to the periplasm to capture another sugar. Such an ATP-driven alternating access model has been strongly supported by biochemical and crystallographic analysis, on this and other ABC transporters [11], [12]. An EPR spectroscopy study has also concluded that liganded-MalE is required for the closure of the nucleotide-binding interface [13]. Yet, despite the long-lasting prevalence of the model, the notion that MalE shuttles back and forth to the membrane to deliver maltose has not always been consistent with earlier genetic analysis. For example, it was expected that the transport constant *K*_t_ would decrease rapidly when MalE concentration increases in the periplasm [14]. In reality, the *K*_t_ decreased only ∼2-fold when the MalE concentration increased more than ∼20-fold [15]. Similarly, the activation of the MalK ATPase was expected to be strong with a MalE variant possessing high affinity for maltose, but instead the mutant showed impaired ability to stimulate transport [16]. It was also surprising that a MalFGK_2_ allele capable to transport lactose was still dependent on MalE for activity, although MalE does not binds lactose [17]. It was then unexpected that excess MalE can inhibit transport when maltose is held at a sub-stoichiometry level [18]. Finally, maltose-loaded MalE was reported to have low affinity for the transporter (50–100 µM) [19]. Since MalE periplasmic concentration depends on maltose, the transport efficiency may be weak at low maltose [20], whereas MalE is to facilitate transport especially at limiting substrate concentration.

In this study, we examined the problem of substrate delivery by measuring the effect of maltose on the stability of the MalE-MalFGK_2_ complex. We employed the nanodisc because the system is well adapted to membrane proteins and transporters [21], [22]. We report that maltose-free MalE binds the P-open transporter with high affinity (*K*_d_ ∼79 nM), whereas at saturating maltose concentration, MalE captures the sugar (with *K*_d_ ∼120 µM) and dissociates from the transporter. The surprising behavior of liganded-MalE was not specific to the disc because the same observations were made in proteoliposomes. The consequence of this maltose-regulated interaction was evaluated *in* vitro and *in vivo*: maltose transport and maltose-dependent MalK ATPase were found maximal when MalE had low affinity for maltose, and minimal when MalE had high affinity for maltose. We conclude that the transporter activity depends on two opposite effects: the capture and transport of maltose by the MalE-MalFGK_2_ complex, and the capture of maltose by MalE leading to its dissociation from MalFGK_2_. Maltose is therefore both substrate and regulator of its own transporter (i.e. homotropic regulator). Similar allosteric mechanism may apply to all ABC importers dependent on a substrate-binding protein similar to MalE.

## Results

### Reconstitution of the maltose transporter in nanodiscs

**Figure 1:**
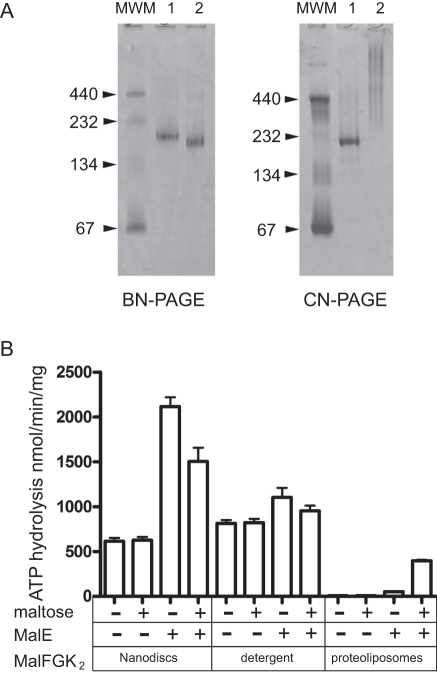
ATPase activity of the MalFGK_2_ complex in nanodiscs. (**A**) The MalFGK_2_ complex in nanodisc (lane 1) and detergent solution (lane 2) was analyzed by BN-PAGE and CN-PAGE followed by Coomassie blue staining of the gel. Molecular weight markers: BSA (67/134 kDa); catalase (232 kDa); ferritin (440 kDa). On CN-PAGE, the MalFGK_2_ complex precipitates as a protein smear at the top of the gel. (**B**) The ATPase activity supported by Nd-MalFGK_2_ was compared to detergent solubilized MalFGK_2_ and MalFGK_2_ in proteoliposomes (2 µM each) at 37°C in the presence of MalE (2 µM) or maltose (1 mM) in TSGM Buffer (50 mM Tris-HCl pH 8, 50 mM NaCl, 5% glycerol, 5 mM MgCl_2_). The reported values were derived from 3 independent experiments.

The MalFGK_2_ complex was reconstituted in nanodiscs using the membrane scaffold protein MSP1D1 [21]. The discs (hereafter termed Nd-MalFGK_2_) were isolated by gel filtration and analyzed by native-PAGE (Figure 1A). The particles were soluble and homogeneous, with a mean diameter of ∼12 nm (+/− 2nm) and an apparent molecular mass of ∼215 kDa, as expected for a properly reconstituted disc (Figure S2). The ATPase activity supported by the assembly was measured at 37°C (Figure 1B and Figure S3). compared to the proteoliposomes, the basal ATPase activity in disc was ∼10 fold higher (∼700 nmol/min/mg), quite similar to that in detergent (Figure 1B). It is proposed that the detergent micelles (or the lack of lipid bilayer) decrease the activation energy barrier of the transporter [23], and this may also be true in nanodiscs (Bao et al., submitted). However, whether in disc, in detergent or in the membrane, MalE increased the rate of ATP hydrolysis (3-fold, 1.3-fold and 4-fold respectively; Figure 1B) [24], [25]. This last result showed that MalE facilitates (or stabilize) the conversion of MalFGK_2_ toward the P-open ATPase active conformation, whether the transporter endogenous ATPase activity is high or low. Surprisingly, in disc and in detergent, a significant inhibition of the ATPase activity was observed in the presence of maltose (Figure 1B). In the absence of a separating membrane, the concentration of maltose around the transporter is constant, and this may slow down maltose release and therefore the ATPase turnover. This scenario is however unlikely because maltose alone did not effect the basal ATPase activity, nor the affinity of the transporter for ATP (Figure 1B and Figure S4). Thus, the sugar negative effect may be on the association of MalE with the transporter.

### Maltose-free MalE binds with a high-affinity to the P-open transporter

**Figure 2:**
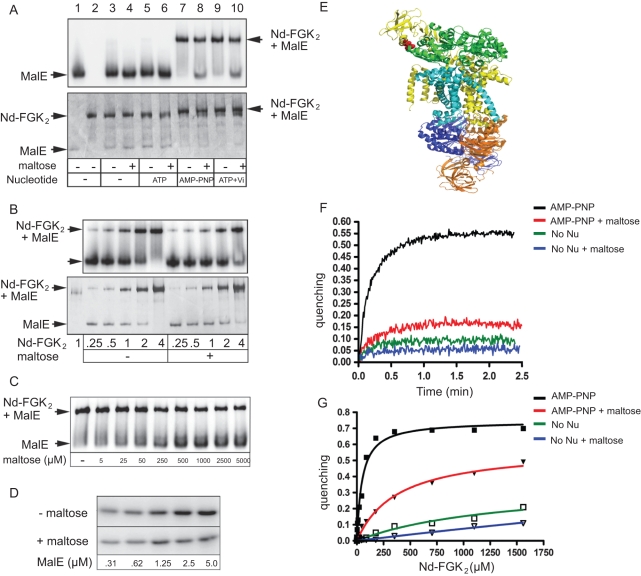
High-affinity binding of MalE to the MalFGK_2_ complex. (**A**) Nd-MalFGK_2_ (4 µM) was incubated with MalE (1 µM) or [^125^I]-MalE (∼10,000 c.p.m., 1 µM) in TSGM buffer containing nucleotides (1 mM) and maltose (1 mM) as indicated. After incubation (10 min, 37°C), samples were analyzed by CN-PAGE followed by Coomassie blue staining (bottom part) and autoradiography (upper part). (**B**) The indicated amount of Nd-MalFGK_2_ was incubated with MalE or [^125^I]-MalE in the presence or absence of maltose (2 mM) in TSGM buffer containing AMP-PNP (1 mM). After incubation (10 min, 37°C), samples were analyzed by CN-PAGE followed by Coomassie blue staining (bottom part) then autoradiography (upper part). (**C**) Nd-MalFGK_2_ (4 µM) was incubated with [^125^I]-MalE (∼10,000 c.p.m., 1 µM) in TSGM buffer containing AMP-PNP (1 mM) and the indicated amount of maltose. After incubation (10 min, 37°C), samples were analyzed by CN-PAGE and autoradiography. (**D**) Nd-MalFGK_2_ (4 µM) was immobilized onto Ni-NTA Sepharose beads and incubated with [^125^I]-MalE (∼10,000 c.p.m., 1 µM) in TSGM buffer containing AMP-PNP (1 mM) in the absence or presence of maltose (1 mM). After incubation (10 min, room temperature), bound MalE was eluted and revealed by SDS-PAGE and autoradiography. (**E**) Structure of the complex MalFGK_2_-(E159Q) with MalE. The position MalE-31 and MalF-177 are indicated in red. (**F**) Time course fluorescence quenching between MalE (20nM) and Nd-MalFGK_2_ (90 nM) in the presence or absence of maltose (1mM) and AMP-PNP (1mM). (**G**) Equilibrium titration of MalE (20 nM) fluorescence quenching with up to 1.5 µM Nd-MalFGK_2_. When the data were fitted to one-site binding equation, the dissociation constant in the presence of AMP-PNP was ∼79 nM. The dissociation constant in the presence of AMP-PNP and maltose was ∼390 nM. When the data were fitted to a competitive one-site binding equation, in which maltose-bound MalE do not bind to the transporter and maltose acts as a competitor, the dissociation constant of transporter-bound MalE for maltose was 127 µM.

To test the above hypothesis, we determined the affinity of MalE for MalFGK_2_ and the effect of maltose on the complex stability. A complex of MalE and MalFGK_2_ can be isolated with non-hydrolysable ATP analogs or in the presence of vanadate. These conditions stabilize the P-open state transporter [7], [26]. Accordingly, MalE and Nd-MalFGK_2_ migrated to different positions on native-PAGE but together in the presence of AMP-PNP or ATP plus vanadate (Figure 2A). However, in the presence of maltose, the binding of MalE to MalFGK_2_ was significantly reduced (Figure 2A, compare lane 7 to lane 8). The negative effect of maltose was further confirmed by titration analysis (Figure 2B and 2C) and pull-down experiments (Figure 2D).

**Table 1:** Dissociation constants of MalE for Nd-MalFGK_2_ determined in the presence or absence of AMP-PNP and maltose.

**Conditions**	***K*d (nM)**
AMP-PNP	79.4 ± 9.3
AMP-PNP+maltose	391.6 ± 52.2
No Nu	NA
No Nu+maltose	NA

The data were collected and analyzed according to Figure 2G.

To determine the binding affinities, we employed an electron transfer-based quenching reaction [27], [28]. MalE cysteine residue position 31 was modified with the oxazine-derivative dye ATTO655, and incubated with Nd-MalFGK_2_ bearing a tryptophan at position MalF-177. These two amino acids are within ∼5Å distance in the MalE-MalFGK_2_ complex structure (Figure 2E, Figure S5, and [7], [29]). In the absence of nucleotide, very little quenching occurred (Figure 2F, green curve), in agreement with an earlier EPR spectroscopy analysis showing that MalE has no measurable affinity to the P-closed transporter (i.e. >50–100 µM) [19]. In contrast, rapid and strong fluorescence quenching occurred with AMP-PNP, confirming that maltose-free MalE binds with high-affinity to the P-open transporter (Figure 2F, black curve). The data were fitted to the one-site ligand binding equation, and the equilibrium affinity of maltose-free MalE for P-open MalFGK_2_ was determined to be ∼79 nM (Table 1, Supporting Information S1 and Figure S1). As above, maltose had a negative effect on the stability of the complex because the binding affinity dropped ∼5-fold to 390 nM (Figure 2G, red curve and Table 1). Interestingly, the binding of MalE to the transporter was still happening at saturating maltose concentration (Figure 2C and 2G). Since MalE is a highly dynamic protein that constantly bind, capture and release maltose [30], it is possible that any maltose-free MalE is immediately captured by the P-open state transporter. In support to the model, the quenching data were best fitted to a competitive ligand binding equation in which maltose-loaded MalE does not bind the transporter at all, whereas transporter-bound MalE has an affinity for maltose around ∼127 µM (Supporting Information S1 and section below).

**Figure 3:**
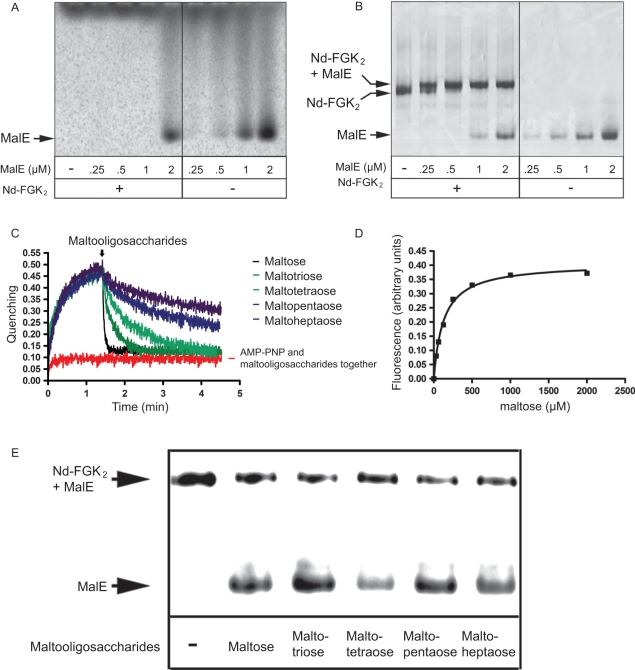
MalE has low affinity for maltose when bound to MalFGK_2_. The indicated amount of MalE was incubated with Nd-MalFGK_2_ (0.5 µM) and [^14^C]-maltose (10 µM, 57 µCi/µmol) in TSGM buffer containing AMP-PNP (1 mM). After incubation (10 min, 37°C), samples were analyzed by CN-PAGE and (**A**) autoradiography or (**B**) Coomassie blue staining. (**C**) The binding of MalE to the transporter was monitored by fluorescence quenching, using MalE (20 nM) and Nd-MalFGK2 (70 nM). At the indicated time (arrow), 1 mM maltooligosaccharides were added to the reaction mixture. (**D**) Equilibrium titration to determine the maltose affinity of the MalE-FGK_2_ complex using MalE (20 nM) and Nd-MalFGK_2_ (70 nM). The derived dissociation constant was 120 µM. (**E**) Nd-MalFGK_2_ (4 µM) was incubated with [^125^I]-MalE (∼10,000 c.p.m., 1 μM) in TSGM buffer containing AMP-PNP (1 mM) and the indicated maltooligosaccharides (1 mM). After incubation (10 min, 37°C), samples were analyzed by CN-PAGE and autoradiography.

**Figure 4:**
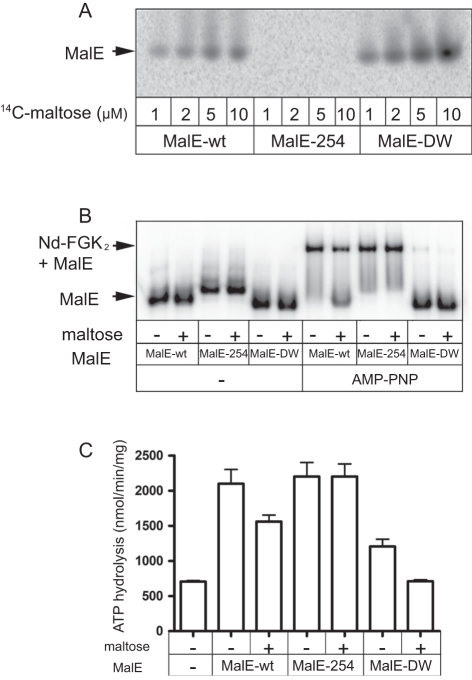
Binding of the MalE mutants to MalFGK2. (A) Wild type MalE and mutants (1 µM each) were mixed with [^14^C]-maltose in TSG buffer. Samples were analyzed by CN-PAGE and autoradiography. (**B**) [^125^I]-labeled MalE and variants were incubated with Nd-MalFGK_2_ (4 µM) in TSGM buffer containing AMP-PNP and maltose (2 mM) as indicated. After incubation (10 min, 37°C), samples were analyzed by CN-PAGE and autoradiography. (**C**) The ATPase activity supported by the MalE mutants (1 µM each) was determined in the presence of Nd-MalFGK2 (2 µM) and maltose (1 mM). The reported values were derived from 3 independent experiments.

### MalE captures maltose and looses affinity for MalFGK_2_

We employed ^14^C-maltose to localize the sugar when incubated with MalE and MalFGK_2_. Free MalE has a relatively high affinity for maltose (*K_d_* ∼2 µM), and native-PAGE can detect this association (Figure 3A, right panel). In contrast, when MalE was bound to the P-open transporter (Figure 3B), ^14^C-maltose was not detected associated with the complex (Figure 3A). Thus, either the binding site on MalE is not accessible to the sugar, or MalE binds the sugar but dissociates from the transporter. To test the two possibilities, ATTO655-labeled MalE was bound to the transporter in the presence of AMP-PNP (Figure 3C, black curve). Upon addition of maltose, there was a rapid loss of fluorescence quenching, indicating the dissociation of MalE from the transporter (Figure 3C, black curve). Using this assay, the maltose affinity of transporter-bound MalE was determined to be ∼120 µM (Figure 3D); a value very similar to that derived from the competitive one-site ligand binding equation (∼127 µM; Supporting Information S1). The result was surprising because the X-ray structure of the MalE-MalFGK_2_ complex did not reveal any accessibility pathway for maltose [7]. Here, oligosaccharides from three (maltotriose) to seven (maltoheptaose) units were able to promote dissociation of the complex (Figure 3C and 3E). The interaction MalE-MalFGK_2_ in the presence of AMP-PNP may be more dynamic than expected, or a path at the protein interface may be large enough to let maltose access MalE. Most important to this analysis, the results showed that in the absence of transport, MalE captures maltose and dissociates from the transporter.

**Figure 5:**
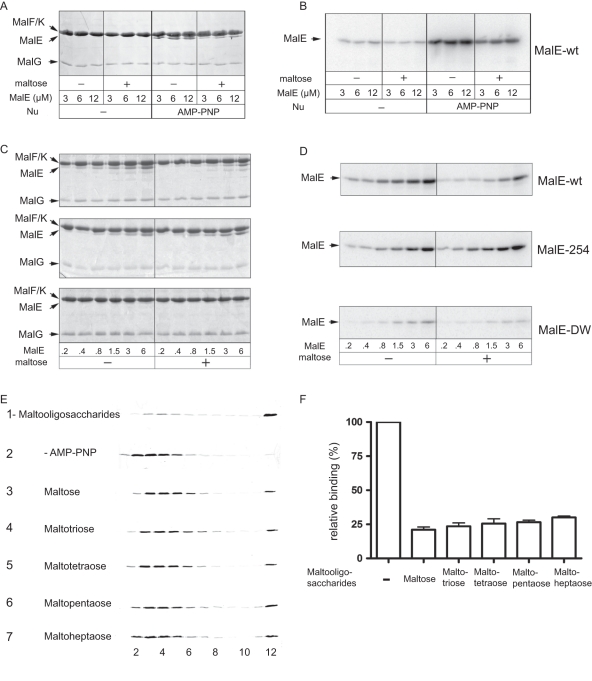
Binding of MalE to the MalFGK_2_ complex in proteoliposomes. (**A**) [^125^I]-MalE was incubated with MalFGK_2_ proteoliposomes (2 µM) in TM buffer (20 mM Tris-HCl pH 8.0, 10 mM MgCl_2_) with or without AMP-PNP (1 mM) and maltose (1 mM) as described in [26]. The fraction of MalE bound to MalFGK_2_ was isolated by ultra-centrifugation. The samples were subjected to SDS-PAGE followed by Coomassie blue. (**B**) Autoradiography of the same gel. (**C**) The co-sedimentation assay was performed using MalE and variants in the presence of AMP-PNP. (**D**) Autoradiography of the same gel. (**E**) MalFGK_2_ in proteoliposomes (10 µM) was incubated with ATTO655-labeled MalE-31C (0.5 µM) in the presence of AMP-PNP. The samples were applied on a sucrose density gradient containing the indicated maltooligosaccharides (1 mM). Equal fractions were collected and analyzed by SDS-PAGE and fluorescence assay. The control experiments showed that MalE did not co-sediment with MalFGK_2_ in the absence of AMP-PNP (sample 2), but very well in the absence of maltose (sample 1). (**F**) Quantification of MalE bound to MalFGK_2_ in proteoliposomes. The amount of MalE bound to MalFGK_2_ without maltooligosaccharides was set to 100%.

### MalE with low affinity for maltose has a high affinity for MalFGK_2_ and *vice versa*

Since the binding of MalE to the transporter was found controlled by maltose, the conformational state of MalE perhaps determine the binding affinity to the transporter. To test this hypothesis, we employed the mutant MalE-A96W/I329W (hereafter termed MalE-DW), which has ∼60 fold stronger affinity for maltose (Figure 4A) [31]. The two mutations, located at the ‘balancing interface’ opposed to the sugar binding site, favor the closed state of MalE even in the absence of maltose, as shown by NMR and SAXS analysis [16], [31]–[33]. We also employed the mutant MalE-254 (mutation D65N) that displays very low affinity for maltose (*K_d_* >1 mM; Figure 4A and Table 2) [34], [35]. The side chain D65 normally creates hydrogen bonds with the sugar hydroxyls [36] and previous fluorescence and UV spectra analysis suggested that MalE-254 does not acquire the characteristic closed-liganded conformation until at least 10 mM maltose [35]. It is thus very likely that MalE-254 would remain in open state at the maltose concentrations used in our assays (Figure 4A). Native-PAGE and ATPase assays were employed to determine the capacity of the two mutants to bind and activate the transporter (Figure 4B and 4C). MalE-DW was mostly unable to associate with Nd-MalFGK_2_ and it supported very little ATPase activity, which was further reduced by maltose (Figure 4B and 4C). In contrast, MalE-254 formed a tight complex with Nd-MalFGK_2_ and the ATPase activity was maximal and independent from maltose, as expected since MalE-254 does not capture the sugar. We therefore concluded that (i) maltose-free MalE facilitates the conversion of MalFGK_2_ toward the ATPase active conformation, (ii) maltose-free MalE binds with high-affinity to the P-open transporter (*K_d_* ∼79 nM), (iii) maltose has access to transporter-bound MalE, and (iv) upon capture of maltose, MalE looses its affinity for the transporter (>50–100 µM).

**Figure 6:**
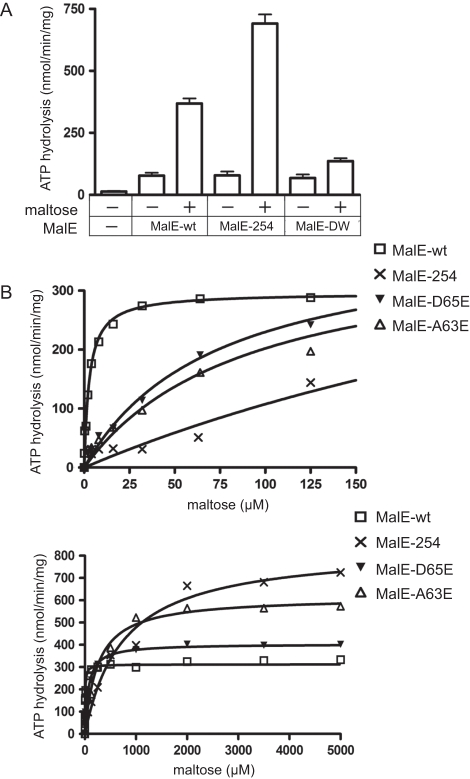
Regulation of the MalK transport ATPase by maltose. (**A**) ATP hydrolysis was measured with MalFGK_2_ proteoliposomes (37°C, 10 min) in the absence or presence of maltose (2 mM) using MalE and variants (2 µM each). (**B**) Steady-state transport ATPase using MalE variants (2 µM each) as a function of the maltose concentration. Left panel and right panel are the same curve but fitted to different x-axis. The data were fitted to the Michaelis-Menten equation to determine the maximal velocity *V_max_* and *K_t_* of the transport ATPase reaction. The calculated values derived from 3 independent experiments are presented in Table 2.

### The results obtained with the nanodisc are confirmed in proteoliposomes

The binding of MalE to MalFGK_2_ in proteoliposomes was assessed by co-sedimentation assays (Figure 5A and 5B, Figure S6). In proteoliposomes, the conformational state of the transporter is shifted toward the P-closed state, which has low ATPase activity and low affinity for MalE [9], [26]. As expected, AMP-PNP stabilized the P-open state and increased the affinity for MalE over 30-fold (Figure 5A and Figure S6 for quantitation). However, as in nanodiscs, the addition of maltose reduced the MalE equilibrium binding affinity by at least ∼3-fold (Figure 5A). Furthermore, the co-sedimentation efficiency of MalE-254 was strong and independent from maltose (Figure 5C and 5D), whereas the co-sedimentation of MalE-DW was poor (∼5-fold less than MalE-wt), and even weaker with maltose. To confirm that maltose had access to MalE when bound to the P-open transporter, the MalE-MalFGK_2_ complex was formed with AMP-PNP, then loaded on a sucrose gradient containing maltodextrins (Figure 5E). In all cases, there was a very obvious dissociation of fluorescent-labeled MalE from the transporter (Figure 5F). The control experiments showed that MalE did not co-sediment with MalFGK_2_ in the absence of AMP-PNP (Figure 5E, sample 2), but did co-sediment very well with AMP-PNP alone (Figure 5E, sample 1). Thus, the binding characteristics of MalE and variants, and the negative effect of maltose, were the same both in nanodiscs and proteoliposomes. In either environment, maltooligosaccharides reduced the equilibrium binding affinity of MalE to the transporter.

**Table 2:** Kinetic parameters of the transport reaction and affinity of MalE variants for maltose.

MalE	*V*_max_ (nmol/min/mg)	*K*_t_ (μM)	*K*_d_ of MalE for maltose (μM)
MalE-wt	320 ± 37	2 ± 0.7	2.8 ± 0.6
MalE-D65E	403 ± 69	78 ± 11	69 ± 15
MalE-A63E	614 ± 34	214 ± 40	161 ± 36
MalE-254	842 ± 47	807 ± 136	3700 ± 412

The data were collected and analyzed according to Figure 6B.

### Dual effect of the sugar on the transporter activity in proteoliposomes

In proteoliposomes, the basal MalK ATPase activity is low (∼10 nmol/min/mg), most likely because the lipid bilayer stabilizes the P-closed state transporter (Figure 1B). The addition of MalE stimulates ∼4-fold the ATPase activity (∼40 nmol/min/mg), and furthermore ∼10-fold in the presence of maltose (Figure 6A and see Discussion on this point). The ATPase measurements were then performed using the two MalE mutants described above. The maltose-dependent ATPase was best served with MalE-254 (∼20 fold stimulation; Figure 6A), even though this mutant did not bind maltose at the concentration used in this assay. In contrast, the mutant MalE-DW, which captured maltose with a high affinity (K_d_ ∼50 nM), was unable to trigger the transporter ATPase activity (Figure 6A). Thus, maltose produced two effects in proteoliposomes: it stimulated the MalK ATPase and it diminished the affinity of MalE for the transporter. This second effect is opposed to the first because it reduces the maltose-dependent ATPase activity.

**Figure 7:**
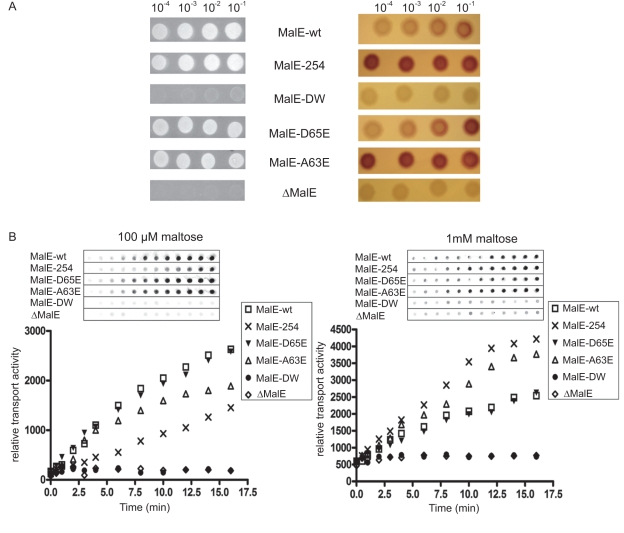
Maltose transport in intact cells. (**A**) Strain HS3309 *(ΔMalE*) transformed with pLH1 encoding for the indicated MalE protein was plated on M9-maltose (left) or MacConkey-maltose (right) agar plate. The color on MacConkey plates reflects maltose transport and fermentation after 10 h. The plasmid vector was used as a negative control. (**B**) The transport assay using [^14^C]-maltose and strain HS3309 was performed as described in materials and methods. At the indicated time, cells were spotted on PVDF membrane and maltose import was detected by autoradiography. The intensity of each dot was determined by using ImageQuant (GE healthcare).

### Maltose is both substrate and regulator of the transporter

To show that maltose produces two opposed effects during transport, the MalK ATPase was determined at various maltose concentrations. In proteoliposomes, the ATPase is coupled to maltose transport in an apparent stoichiometric manner [37], [38]. With MalE-wt, the transport constant was low (*K_t_* ∼2 µM) and the maximal velocity was reached as soon as the maltose concentration reached ∼25 µM (Figure 6B). With MalE-254, the transport constant was high (∼800 µM) and the transport ATPase was quasi-linear until ∼1 mM maltose (Figure 6B). Most strikingly, the maximal transport velocity was hardly reached with this mutant. At 5 mM maltose, the transport ATPase supported by MalE-254 was almost 3-fold higher than with the wild type (Figure 6B and Table 2). Since the mutant MalE-254 is unable to capture the sugar, the transporter ATPase activity was dictated by the maltose concentration. To the opposite, the mutant MalE-DW could barely sustain any maltose-dependent ATPase activity, as expected since the mutant captures maltose and is unable to bind the transporter. We constructed two additional MalE mutants with intermediate affinity for maltose (MalE-D65E and MalE-A63E; Figure 6B and ate is inversely proportional to the affinity of MalE for maltose.

**Figure 8:**
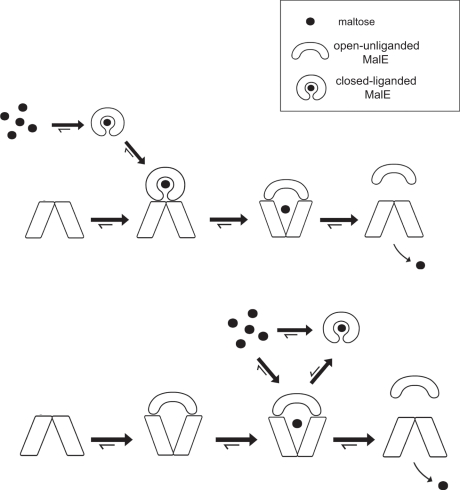
Models of maltose transport. (**A**) In the conventional model, MalE captures maltose with high affinity (K_d_ ∼2 µM), then associates with the P-closed transporter with low affinity (*K*_d_ ∼50–100 µM). The association triggers the P-open state, the opening of MalE and the release of maltose in the translocation cavity. The transporter returns to the P-closed state upon ATP hydrolysis. MalE dissociates and return to the periplasm. (**B**) In the auto-regulation model, MalE binds with high affinity (*K*_d_ ∼79 nM) to the P-open ATPase active transporter. Maltose is then captured by the complex of MalE-MalFGK_2_ (*K*_d_ ∼120 µM) and rapidly transported (*K*_t_ ∼2 µM) upon hydrolysis of ATP. When the concentration of maltose exceeds the import capacity, MalE acquires its closed-liganded conformation and looses affinity for MalFGK_2_. This regulation limits the maximal rate of transport (“transporter set point”). The function of closed-liganded MalE is to retain maltose in the periplasm (“retention effect”).

### Consequence of an unregulated maltose transport in intact cells

The work above allowed us to predict that maltose transport will be highest in bacteria expressing MalE-254. In contrast, maltose transport will be severely compromised in bacteria expressing MalE-DW. To confirm the prediction, maltose utilization was tested on MacConkey media (Figure 7A; Figure S8) and maltose accumulation was measured using ^14^C-maltose (Figure 7B). At saturating maltose concentration (i.e. 1 mM, equivalent to 0.04%), MalE-254 was the most proficient mutant for the transport and the fermentation of the sugar. In contrast, MalE-DW was unable to support cellular growth. Clearly, the high-affinity capture of maltose by MalE-DW was inhibiting transport and inversely, maltose transport was most effective with MalE-254 because the protein is unable to capture the sugar. We also tested the transport activity at sub-saturating maltose concentration (e.g. 100 µM). Previous microbiological work indicated that bacterial growth is slower at maltose concentration below 1 mM [34], [39]. At this limiting sugar concentration, the maltose import was better with MalE-wt compared to MalE-254 (Figure 7B). The result was expected because transporter-bound MalE increases the affinity for maltose, and thus the efficiency of transport when the substrate is limiting in the environment. Accordingly, the transport constant *K_t_* obtained with MalE-wt is ∼2 µM whereas the *K_t_* for MalE-254 is ∼800 µM (Table 2).

## Discussion

In the conventional model, closed-liganded MalE binds and activates the P-closed transporter. The binding triggers a series of ATP-driven conformational changes that eventually leads to the opening of MalE, release of maltose and transport across the membrane (Figure 8). Over the last twenty years, the different steps of the model have been analyzed in details at the biochemical, biophysical and structural levels [8]. Yet, the binding affinity of maltose-free MalE has never been characterized and the effect of maltose on the stability of the MalE-MalFGK_2_ complex has never been reported. Here, we confirm that closed-liganded MalE has weak affinity for MalFGK_2_ (*K_d_* >50–100 µM), but we show that open-unliganded MalE possesses nanomolar affinity for the P-open transporter (*K_d_* ∼79 nM). In addition, we show that maltose can access transporter-bound MalE (K_d_ ∼120 µM) whereas, in the absence of maltose uptake, MalE captures the sugar (*K_d_* ∼2 µM) and dissociates from the transporter (*K_d_* >50 µM). The knowledge of the binding affinities leads us to propose a different model, in which MalE is permanently bound to the transporter to create a low-affinity maltose-binding site. If maltose is not immediately transported, MalE acquires a closed-liganded conformation and dissociates from the transporter (Figure 8). We justify below the reasons for this novel model and the mechanistic and physiological implications.

First, in proteoliposomes. Our results show that the maximal ATPase activity and transport velocity are inversely proportional to the affinity of MalE for maltose. If maltose was to cause MalE to activate the transporter, the MalK ATPase at saturating maltose concentration (V_max_) should be independent from the affinity of MalE for maltose. For example, a variant with low affinity would support as much ATPase provided the sugar concentration is sufficiently high. Reciprocally a variant with high affinity would support the same maximal ATPase but at low concentration of maltose. The results from ATPase assays (Figure 6) and the maltose transport *in vivo* (Figure 7) are not consistent with such model. Furthermore, the binding assays in proteoliposomes show that maltose decreases the equilibrium affinity of MalE to the transporter, whether in the P-open or P-closed state (Figure 5 and Figure S6). The current model is therefore insufficient to explain the data. Instead, we believe that a model in which MalE is bound to the transporter to facilitate the capture of maltose, but dissociates from the transporter when the substrate concentration increases, can explain why the maximal velocity depends on MalE affinity for maltose. A variant with low affinity would display lower transport rate (i.e. higher *K_t_*) but remain bound to the transporter when the substrate concentration increases, allowing for a higher maximal rate of transport. In contrast, a variant with high affinity for maltose would capture the sugar and dissociate from the transporter, hence lowering the maximal rate of transport. Our results (Figure 6 and Figure 7), as well as those in Wandersman *et al*. (1979) and Gould et *al*. (2009) which describe the behavior of the mutant MalE-254 and MalE-DW respectively, concur with this analysis. The model is also consistent with the observation that excess MalE can inhibit transport when maltose is held at a sub-stoichiometry level [18]. In the later case, all maltose molecules would be captured by excess MalE and away from the transporter.

Second, in nanodiscs. We find that maltose decreases the affinity of MalE for MalFGK_2_ and decreases the MalE-dependent MalK ATPase activity. According to the former model, maltose should instead stimulate the MalK ATPase, or at least leaves it unchanged. We believe that the observed decrease of MalK ATPase activity can be explained by the negative effect of maltose on the MalE-MalFGK_2_ interactions. In support to this model, using a fluorescence-based binding assays (Figure 2), we were able to show that maltose shifts the binding equilibrium toward the dissociation of MalE from the transporter (Figure 3), and therefore toward the diminution of the MalK ATPase. The binding assays also revealed that transporter-bound MalE is accessible to maltose and longer maltooligosaccharides (Figure 3 and Figure 5). This last observation was surprising because the atomic structure of the MalE-MalFGK_2_ complex did not reveal a sugar accessibility pathway at the protein interfaces. It cannot be excluded that protein crystallography may have suppressed some otherwise transient interactions, such as those detected by molecular dynamics simulations [40]. Here, the affinity of the complex MalE-MalFGK_2_ for maltose was estimated at ∼120 µM. In the absence of transport, this maltose concentration would lead to 50% dissociation of MalE from the transporter. It is important to note that our results do not exclude the possibility that transporter-bound MalE binds maltose just before MalE associates tightly with the P-open transporter. However, if MalE was to capture maltose (and thus acquires a closed-state conformation), MalE would dissociate from the transporter and return to the periplasm (Figure 8).

The interplay between MalE, maltose and MalFGK_2_ is complicated by the dynamic nature of MalE that constantly binds, captures and releases maltose [30], [41]. Based on ATPase measurements, the affinity of liganded-MalE for the transporter would be considered significantly high (*K_m,app_* ∼14 µM; Figure S7 and Gould *et al*, 2009). When interpreting the value however, one should remember that liganded-MalE spontaneously releases maltose [30], whereas maltose-free MalE binds the transporter with high-affinity. The spontaneous release of the ligand would explain why saturating maltose does not abolish the binding of MalE to the P-open transporter (Figure 2 and Figure 5). The same phenomena may occur during histidine transport because the binding of HisJ to HisQM is reduced, but only 3-fold in the presence of saturating amount of histidine [42]. In fact, the modest affinity of MalE and HisJ for their ligands (µM range) may be essential to allow sufficiently influx of these nutrients even at saturating environmental concentration. In contrast, for the vitamin B_12_ transporter, the substrate binding protein BtuF binds its ligand with very high affinity (*K_d_* ∼15 nM). In that case, the interaction between BtuF and BtuCD is dramatically reduced by saturating amount of vitamin B_12_ (∼10^5^ fold) [43]. Thus, even though type I and type II ABC importers (i.e. MalEFGK_2_ and BtuCDF) have different membrane domain and substrate binding protein structures, the regulatory effect of the substrate may be similar. In this context, it is particularly interesting that low-affinity and high-affinity transporter for a same substrate -molybdate- can exist in the same cell [44], [45]. Perhaps the high-affinity molybdate transporter would decrease activity as the molybdate concentration increases. The cell would then use the low-affinity transporter system in order to maintain constant molybdate uptake.

In conclusion, the activity of a membrane transporter usually depends on two factors: the affinity of the transporter for the substrate and the velocity of the transport reaction. For MalE-FGK_2_, transporter-bound MalE controls the affinity and ATP the velocity. We show here that maltose also contributes to the transport kinetics. This negative regulation may be crucial for the cell because ABC importers are unidirectional and can achieve (at least in theory) very high concentration gradients, either toxic or consuming unnecessarily the metabolic energy. Homotropic allosteric regulation represents a simple way to limit transport when the environmental substrate concentration is high. Why MalE is twenty-fold more abundant than the transporter in maltose-induced cells is still not entirely clear [15], [46]. It has been proposed that excess MalE ensures that the periplasmic maltose concentration varies more slowly than the outside, a mechanism termed “retention effect”, especially important during bacterial chemotaxis [42], [47]. It has also been proposed that the high MalE concentration may ensure the hoping of maltose from MalE to MalE to facilitate transport across the gel-like environment of the periplasm [42], [48]. It is also possible that large pool of MalE may serve to buffer the negative effect of maltose because a fraction of ligand-free MalE would always be available to bind the transporter even at saturating maltose concentration. All these possibilities remain to be tested.

## Materials and Methods

### Production and purification of MalE and MalFGK_2_

The genes *MalF, MalG* and *MalK* were separately amplified from the *E. coli* K12 genome by PCR, and placed in tandem in pBAD22 plasmid [49]. A His_6_-tag was inserted at the C-terminus of MalK (yielding p22-FGKhis). The gene encoding for the mature part of MalE was cloned into pBAD33, yielding plasmid p33-MalE. Mutations were introduced by PCR-site directed mutagenesis and all constructs were verified by DNA sequencing. Overproduction of MalFGK_2_ was performed using *E. coli* strain BL21. Briefly, 12L of LB medium containing ampicillin (100 µg/ml) were inoculated with an overnight culture. At OD_600_ ∼ 0.5, plasmid expression was induced with 0.2% (w/v) arabinose. After 3 h, cells were collected in TSG buffer (50 mM Tris-HCl, pH 8; 100 mM NaCl; 10% glycerol) containing 0.01% PMSF, and lysed through a French Press (8,000 psi, twice). After low speed centrifugation (5,000×g, 10 min), the membrane fraction was isolated by ultracentrifugation (100,000, 1 h, 4°C) and resuspended in buffer B (50 mM Tris-HCl, pH 8; 5 mM MgCl_2_, 20% glycerol). Membranes (5 mg/ml) were incubated with 1% n-dodecyl-β-D-maltoside (DDM) with gentle shaking (3 h, 4°C). The solubilized proteins were isolated by ultracentrifugation (100,000× g, 1 h, 4°C) and applied onto a Ni-NTA Sepharose column (10 ml resin) equilibrated in buffer B containing 0.01% DDM (buffer C). After intensive washes (10 column volume in buffer C), proteins were eluted with a gradient of imidazole (0–600 mM) in buffer C. The isolated MalFGK_2_ complex was further purified by gel filtration (Superdex 200 HR10/30) in buffer C. Overproduction of MalE was as described for MalFGK_2_, excepted the antibiotic was chloramphenicol (50 µg/ml). Cells were collected in buffer D (50 mM Tris-HCl pH 8.8; 10% glycerol) and lysed through a French Press (8,000 psi, twice). After ultracentrifugation (100,000×g, 1 h, 4°C), the supernatant was applied onto a Resource Q column (1 ml) equilibrated in buffer D. Proteins were eluted with a gradient of NaCl (0–1000 mM). The protein fractions containing MalE were pooled and denatured with 6M Guanidine-HCl. Protein refolding was performed by dialysis with 3 changes of TSG buffer (50 mM Tris-HCl, pH 8; 100 mM NaCl; 10% glycerol).

### Reconstitution of the MalFGK_2_ complex in nanodiscs

The membrane scaffold MSP1D1 was obtained from the Sligar laboratory [21]. *E. coli* total lipids (Avanti polar lipids) were dissolved in chloroform and dried under a steam of nitrogen. The lipids were resuspended in TSG buffer containing 0.5% DDM. A typical reconstitution experiment involved mixing together the MalFGK_2_ complex, the MSPs and the solubilized lipids at a molecular ratio of 1∶3∶60 in TSG buffer containing 0.1% DDM. Detergent was slowly removed with BioBeads (1/3 volume) and gentle shaking (overnight, 4°C). The reconstituted discs were centrifuged (20 min; 100,000× g), then injected onto a high-pressure packed Superdex 200 HR10/20 column equilibrated in TSG buffer. The fractions containing the Nd-MalFGK_2_ particles were pooled and stored at −80°C.

### Reconstitution of the MalFGK_2_ complex in proteoliposomes

MalFGK_2_ (50 µg) and *E. coli* total lipids (500 µg) were mixed together in RS buffer (20 mM Tris-HCl, pH 8; 100 mM NaCl;10% glycerol; 1 mM DTT; 0.15% DDM) at a lipid:protein ratio (mg/mg) of 10∶1. Detergent was slowly removed with BioBeads (1/5 volume) under gentle shaking (overnight, 4°C). The reconstituted proteoliposomes were harvested by centrifugation (100,000× g, 1 h, 4°C), resuspended in 20 mM Tris-HCl (pH 8) and frozen in liquid nitrogen. Proteoliposomes were sonicated (2 sec, 3 pulses) before use.

### Sedimentation and pull-down assays

For the sedimentation assays, the MalFGK_2_ proteoliposomes (2 µM) were incubated with MalE in 20 mM Tris-HCl (pH 8) containing 10 mM MgCl_2_ for 10 min at 37°C. The sample was diluted 25-fold in 20 mM Tris-HCl (pH 8), collected by ultracentrifugation (100,000× g, 1 h) and resuspended in 20 mM Tris-HCl (pH 8). Samples were analyzed by SDS-PAGE followed by Coomassie blue staining and autoradiography. For the pull-down assays, His_6_-tagged Nd-MalFGK_2_ particles were immobilized onto Ni-NTA resin (10 µL per sample) in TSGM buffer. Samples were then incubated with the indicated amount of [^125^I]-labeled MalE (10 min at room temperature). Unbound MalE was removed by washing the resin 3 times in TSGM buffer. The proteins were eluted in TSGM buffer containing 500 mM imidazole and analyzed by SDS-PAGE and autoradiography.

### Fluorescence labeling

MalE (3 mg/ml) in 500 µL TSG buffer was incubated five-fold molar excess of ATTO-655 (Atto-Tec, GmbH) for 12 h at room temperature in the dark. The labeled protein was separated from excess dye by Superose 6 gel filtration chromatography. The labeling efficiency was determined at different protein concentration by absorbance spectroscopy (663 nm) using the extinction coefficient of 1.25×10^5^ M^−1^cm^−1^. The typical ratio of fluorophore to MalE was 0.8, indicating very efficient labeling.

### Fluorescence spectroscopy

Fluorescence was recorded on a Cary Eclipse spectrofluorometer at 25°C. The affinity of MalE for maltose was determined by intrinsic fluorescence quenching [32]. Excitation and emission wavelength were 280 nm and 350 nm, respectively (10 nm slit widths). Fluorescence quenching of ATTO655-labeled MalE was recorded with excitation and collection wavelengths at 640 nm and 681 nm, respectively (10 nm slit width). The fluorescence emission was monitored over time and the signal was allowed to equilibrate after each addition for 180 s. The fluorescence quenching efficiency (E) was calculated according to the following equation, 
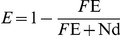
 where *F*_E_ and *F*_E+Nd_ are the fluorescence intensities of ATTO 655-labeled MalE in the presence and absence of Nd-MalF_177w_GK_2_.

### *In vivo* maltose transport assays

Cell cultures were harvested during the late exponential phase of growth, washed twice with M63 salts, and resuspended in the same medium containing 100 µg/ml chloramphenicol to an OD_600_ ∼ 0.5. Each transport assay contained 200 µl of cells and 200 µl of M63 medium supplemented with [^14^C]-maltose at a final concentration of 100 µM (5.7 µCi/μmol) or 1 mM (0.57 µCi/*μ*mol). At the indicated time after incubation at room temperature, 20 μl aliquots of cells were loaded onto a Bio-dot apparatus (Bio-Rad) and washed with 500 μl of M63 medium. The membrane filters were dried and analyzed by autoradiography. The density of each dot was determined by using ImageQuant (GE Healthcare).

### Other methods

The MalFGK_2_ ATPase activity was determined by monitoring the release of inorganic phosphate using photo-colorimetric method [50]. Linear gradient blue-native (BN) and colorless-native (CN) gel electrophoresis was performed as described [51]. Sucrose gradient analysis was performed as described [52]. MalE was iodinated using the Iodogen reagent (Pierce-Thermo Scientific). The specific activity of [^125^I]-MalE was ∼2×10^5^ c.p.m./μg. The detection of [^125^I]-MalE and ^14^C-labeled maltose (57 µCi/μmol, Molecular probes) was performed using a phosphor-imager scanner.

## Supporting Information

Supporting Information S1 **Equations used in this study.**
(DOC)

Figure S1 **Relative binding of MalE to MalFGK_2_ in the presence of maltose.** The amount of MalE bound to Nd-MalFGK_2_ in the absence maltose was set at 100% for each of the Nd-MalFGK_2_ concentration employed. The experimental results obtained in the presence of maltose were plotted next to the calculated values derived from the one-site competitive binding equation.
(EPS)

Figure S2 **Dynamic light scattering analysis of the Nd**-**MalFGK_2_ particles.** The reconstituted MalFGK_2_ complex in Nanodiscs was purified by gel filtration (A) and analyzed by dynamic light scattering using a Dawn-Heleos multi-angle detector (Wyatt Technology). More than 98% of the NdFGK_2_ particles have a diameter of ∼12.5 nm (+/−2 nm) and an apparent molecular weight is 215 kDa.
(EPS)

Figure S3 **The steady-state ATPase of Nd-MalFGK_2_ in the presence of MalE-wt.** The basal ATPase activity of the Nd-MalFGK_2_ complex was subtracted from all measurements. Each value is the average of three different measurements, with standard deviations shown as error bars (some smaller than symbols).
(EPS)

Figure S4 **The affinity of MalFGK_2_ for the nucleotide.** The rate of ATP hydrolysis was determined as a function of the ATP concentration using Nd-MalFGK_2_ (black), Nd-MalFGK_2_ plus MalE (yellow), and Nd-MalFGK_2_ plus MalE and maltose (red). The data were fitted to the Michaelis-Menten equation to calculate the apparent *K*_m_ for ATP.
(EPS)

Figure S5 **ATPase activity of MalF_177W_GK_2_ in the presence of Atto655-MalE.** The ATPase activity of MalF_177W_GK_2_ reconstituted in Nanodiscs and proteolipsomes (2 µM in each case) was measured in the presence of Atto655-MalE (1 µM) and maltose (1 mM). The results show that the modified proteins function like their wild type counterparts.
(EPS)

Figure S6 **Measure of the complex formation between MalE and MalFGK_2_ reconstituted in proteoliposomes.** The density of the bands detected on Figure 5A was determined after scanning by the ImageQuant software. The value obtained in the presence of 12 µM MalE and AMPPNP was normalized to 100%.
(EPS)

Figure S7 **Steady-state transport ATPase supported by MalE and variants.** The ATPase activity of MalFGK_2_ proteoliposomes was measured using various concentrations of MalE and variants in the presence of maltose (2 mM). The calculated apparent *K_m_* for MalE-wt, MalE-254 and MalE-DW were ∼14 µM, ∼4 µM and ∼25 µM, respectively. These *K_m,app_* values do not reflect the real affinity of MalE for the transporter for two reasons: (*i*) a fraction of MalE has captured maltose and therefore unavailable to bind to the transporter and (*ii*) the transporters oscillate between two conformations of different affinity for MalE. The current model of maltose delivery to the transporter implies that the maltose concentration that produces half-maximal transport (*K_t_* ∼2 µM) depends on the affinity of MalE for maltose in solution (*K_d_* ∼2 µM*)*. In other words, the binding of MalE to MalFGK_2_ should not be rate-limiting in transport process. Instead, the results show that the apparent affinity of MalE for the transporter in the presence of maltose (*K_m,app_* ∼14 µM) is lesser than the affinity of the transporter complex for maltose.
(EPS)

Figure S8 **Maltose utilization measured in MacConkey liquid media.** MacConkey-maltose broth was inoculated with strain HS3309 *(ΔMalE*) transformed with plasmid pLH1 encoding for the indicated MalE protein. After 10 h at 37°C, cells were collected and the absorbance of the supernatant was measured. The quenching of the light absorbance at 590 nm reflects the degree of maltose transport and utilization.
(EPS)
