# Supplementary material for: Discovery of an Auto-Regulation Mechanism for the Maltose ABC Transporter MalFGK2
Source: PLoS One. 2012 Apr 17;7(4):e34836. doi: 10.1371/journal.pone.0034836 (PMC3328499; doi:10.1371/journal.pone.0034836)
Supplement: Supporting Information S1 — Equations used in this study. (DOC) [file pone.0034836.s001.doc]

**Supporting information**

**One-site ligand binding**. The quenching of fluorescent ATTO655-MalE by Nd-MalF_177w_GK_2_ was used as a kinetic measure for complex formation. The data were fitted to one-site ligand binding equation, defined as:

where [B] is the amount of MalE bound to Nd-MalFGK_2_, Bmax is the maximal binding capacity of MalE and [L] is the Nd-FGK_2_ concentration.

**Competitive one-site ligand binding.** Maltose and Nd-MalFGK_2_ are competing for the binding to MalE. The data (Fig. 2F, red curve) were fitted to a competitive one-site binding equation, defined as:

where [B] is the amount of MalE bound to Nd-FGK_2_, Bmax the total binding capacity of Nd-MalFGK_2_, [L] the Nd-MalFGK_2_ concentration, [I] the maltose concentration, *K*_d_ the affinity of MalE for Nd-MalFGK_2_ and *K*_I_ the competitive affinity of MalE for maltose. When the *K*_d_ value is 79nM, fitting the data (Figure 2G, red curve) into the equation give the *K*_I_ value of 127µM.

In the figure S1, we show that the competitive ligand binding equation above is consistent with the values measured for the binding of MalE to Nd-FGK_2_ in the presence or the absence of maltose when the *K*_I_ value is fixed at 127µM.
